# Supplementary material for: UPR-Induced miR-616 Inhibits Human Breast Cancer Cell Growth and Migration by Targeting c-MYC
Source: Int J Mol Sci. 2023 Aug 22;24(17):13034. doi: 10.3390/ijms241713034 (PMC10487498; doi:10.3390/ijms241713034)
Supplement: Supplementary file 1 [file ijms-24-13034-s001.zip › Supplementary Figures MIR616 IJMS-23 SG edit.pptx]

## Slide 1
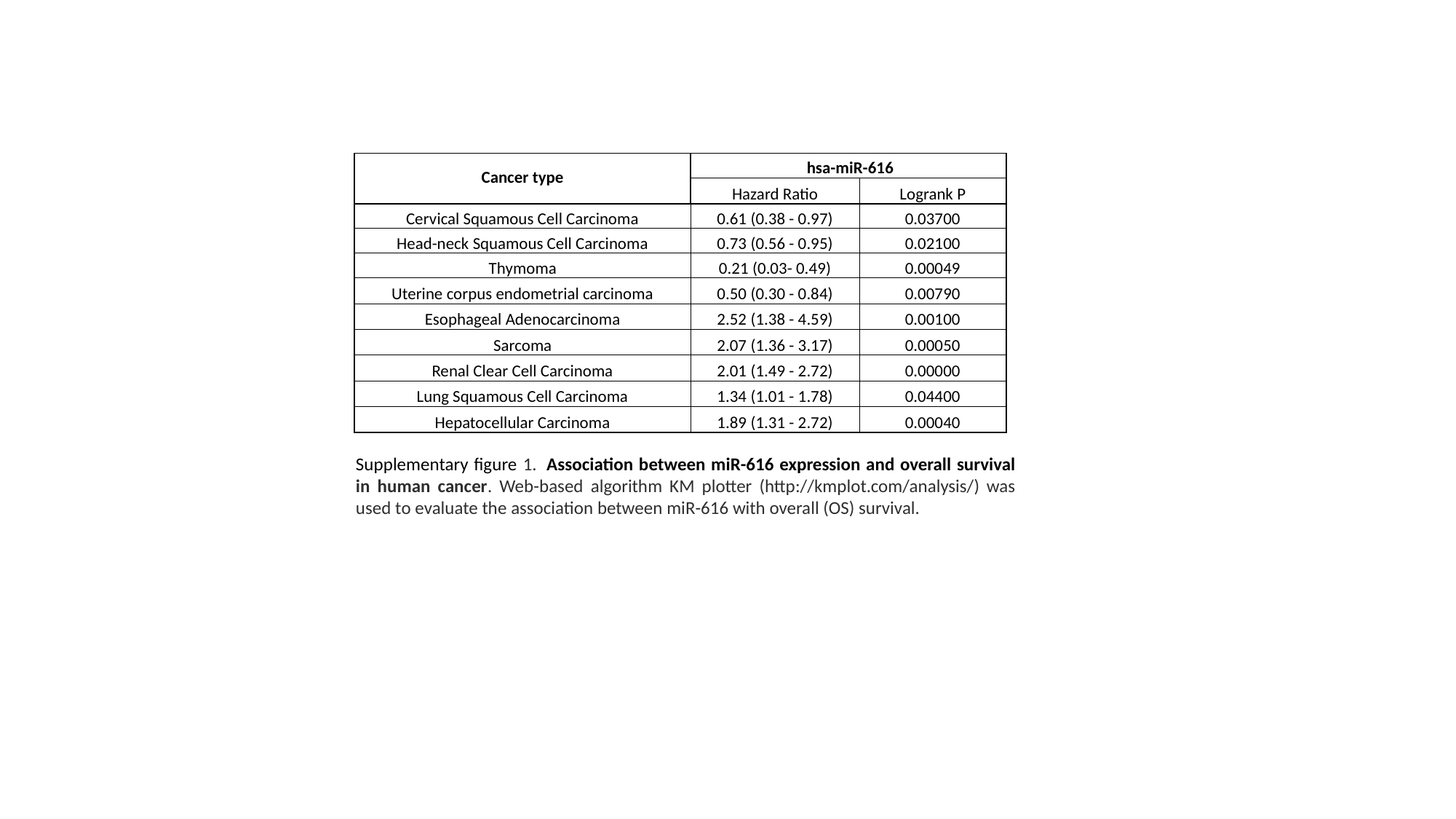

| Cancer type | hsa-miR-616 | |
| --- | --- | --- |
| | Hazard Ratio | Logrank P |
| Cervical Squamous Cell Carcinoma | 0.61 (0.38 - 0.97) | 0.03700 |
| Head-neck Squamous Cell Carcinoma | 0.73 (0.56 - 0.95) | 0.02100 |
| Thymoma | 0.21 (0.03- 0.49) | 0.00049 |
| Uterine corpus endometrial carcinoma | 0.50 (0.30 - 0.84) | 0.00790 |
| Esophageal Adenocarcinoma | 2.52 (1.38 - 4.59) | 0.00100 |
| Sarcoma | 2.07 (1.36 - 3.17) | 0.00050 |
| Renal Clear Cell Carcinoma | 2.01 (1.49 - 2.72) | 0.00000 |
| Lung Squamous Cell Carcinoma | 1.34 (1.01 - 1.78) | 0.04400 |
| Hepatocellular Carcinoma | 1.89 (1.31 - 2.72) | 0.00040 |
Supplementary figure 1.  Association between miR-616 expression and overall survival in human cancer. Web-based algorithm KM plotter (http://kmplot.com/analysis/) was used to evaluate the association between miR-616 with overall (OS) survival.

## Slide 2
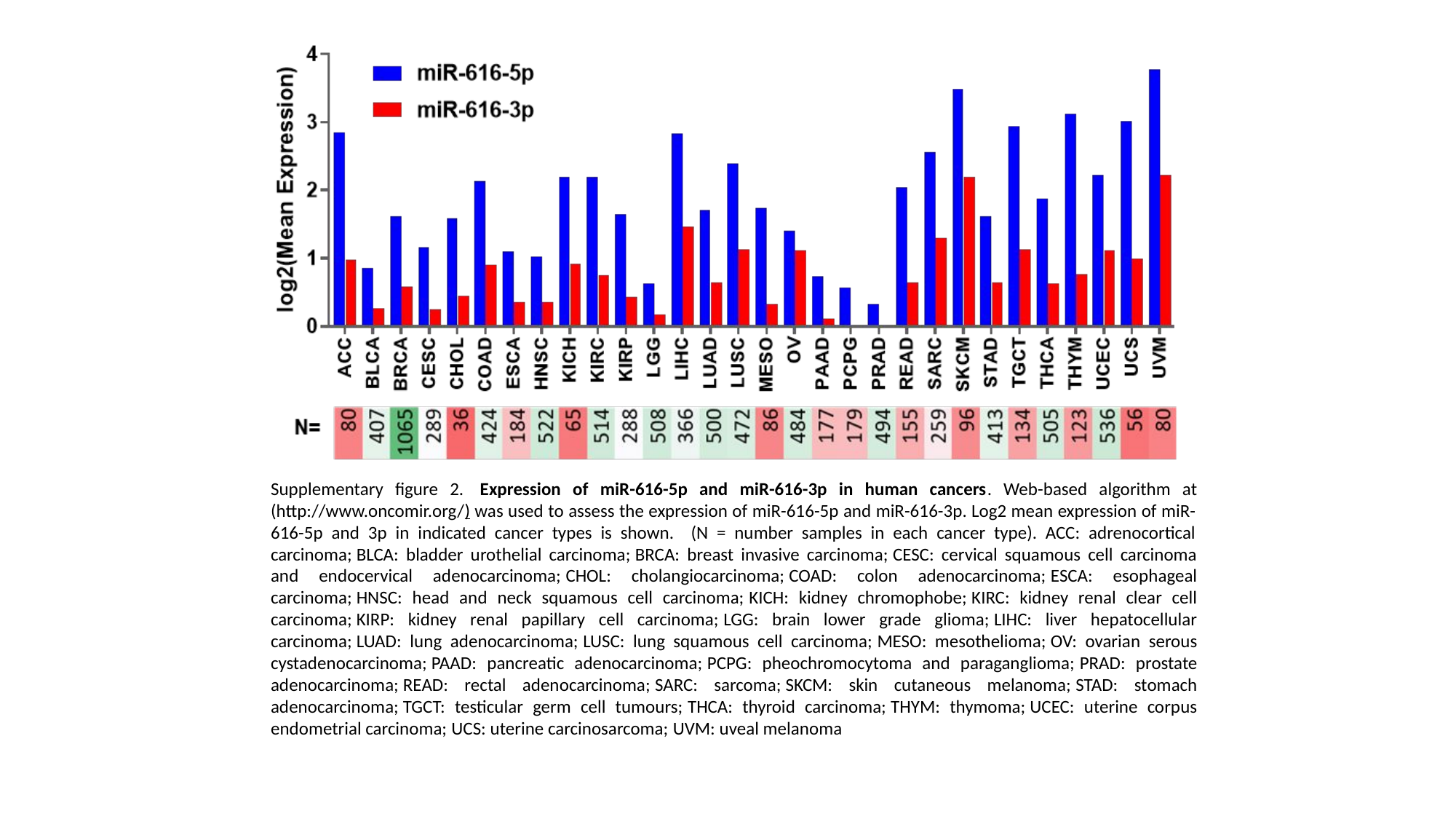

Supplementary figure 2.  Expression of miR-616-5p and miR-616-3p in human cancers. Web-based algorithm at (http://www.oncomir.org/) was used to assess the expression of miR-616-5p and miR-616-3p. Log2 mean expression of miR-616-5p and 3p in indicated cancer types is shown. (N = number samples in each cancer type). ACC: adrenocortical carcinoma; BLCA: bladder urothelial carcinoma; BRCA: breast invasive carcinoma; CESC: cervical squamous cell carcinoma and endocervical adenocarcinoma; CHOL: cholangiocarcinoma; COAD: colon adenocarcinoma; ESCA: esophageal carcinoma; HNSC: head and neck squamous cell carcinoma; KICH: kidney chromophobe; KIRC: kidney renal clear cell carcinoma; KIRP: kidney renal papillary cell carcinoma; LGG: brain lower grade glioma; LIHC: liver hepatocellular carcinoma; LUAD: lung adenocarcinoma; LUSC: lung squamous cell carcinoma; MESO: mesothelioma; OV: ovarian serous cystadenocarcinoma; PAAD: pancreatic adenocarcinoma; PCPG: pheochromocytoma and paraganglioma; PRAD: prostate adenocarcinoma; READ: rectal adenocarcinoma; SARC: sarcoma; SKCM: skin cutaneous melanoma; STAD: stomach adenocarcinoma; TGCT: testicular germ cell tumours; THCA: thyroid carcinoma; THYM: thymoma; UCEC: uterine corpus endometrial carcinoma; UCS: uterine carcinosarcoma; UVM: uveal melanoma

## Slide 3
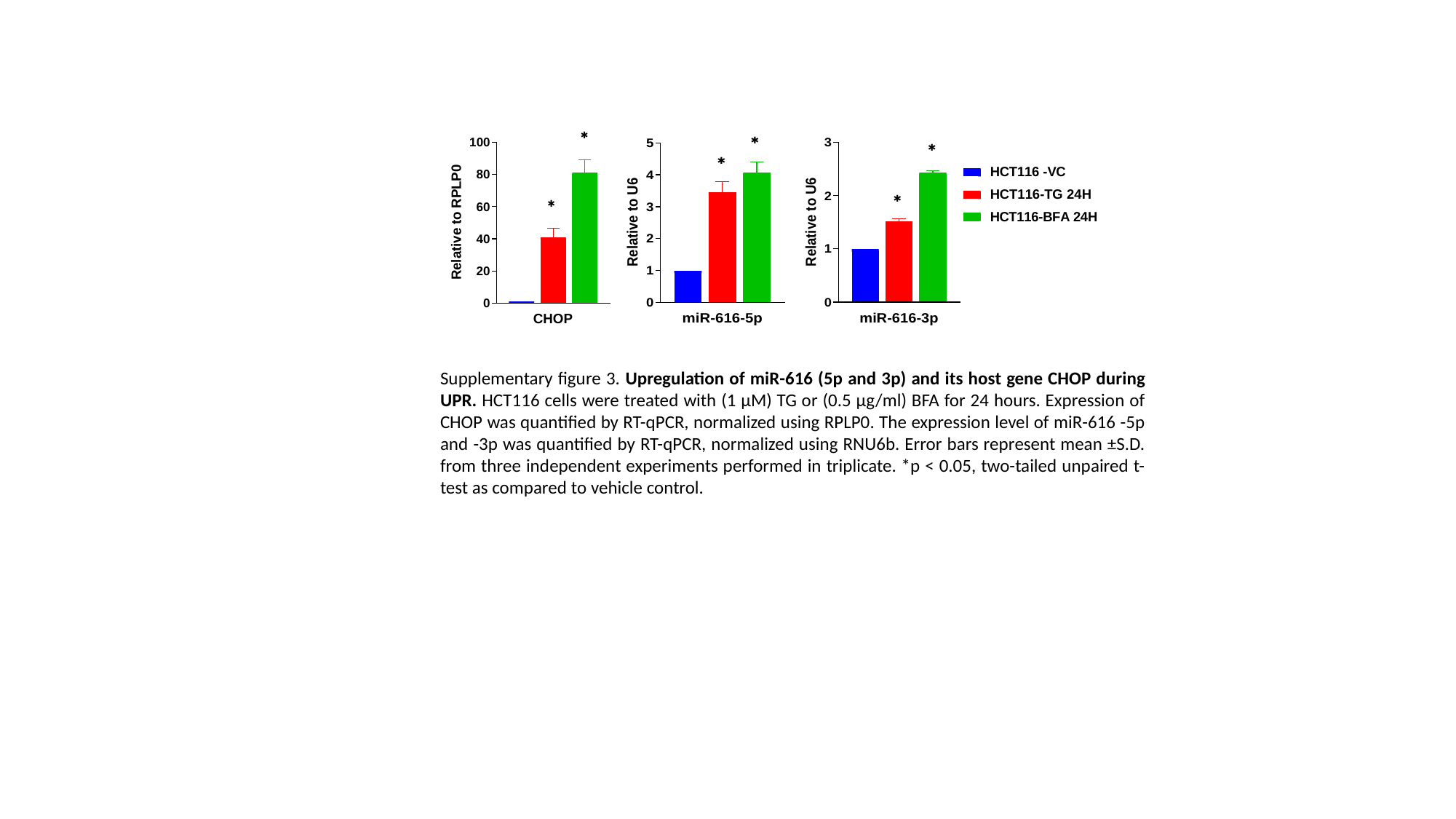

Supplementary figure 3. Upregulation of miR-616 (5p and 3p) and its host gene CHOP during UPR. HCT116 cells were treated with (1 µM) TG or (0.5 μg/ml) BFA for 24 hours. Expression of CHOP was quantified by RT-qPCR, normalized using RPLP0. The expression level of miR-616 -5p and -3p was quantified by RT-qPCR, normalized using RNU6b. Error bars represent mean ±S.D. from three independent experiments performed in triplicate. *p < 0.05, two-tailed unpaired t-test as compared to vehicle control.

## Slide 4
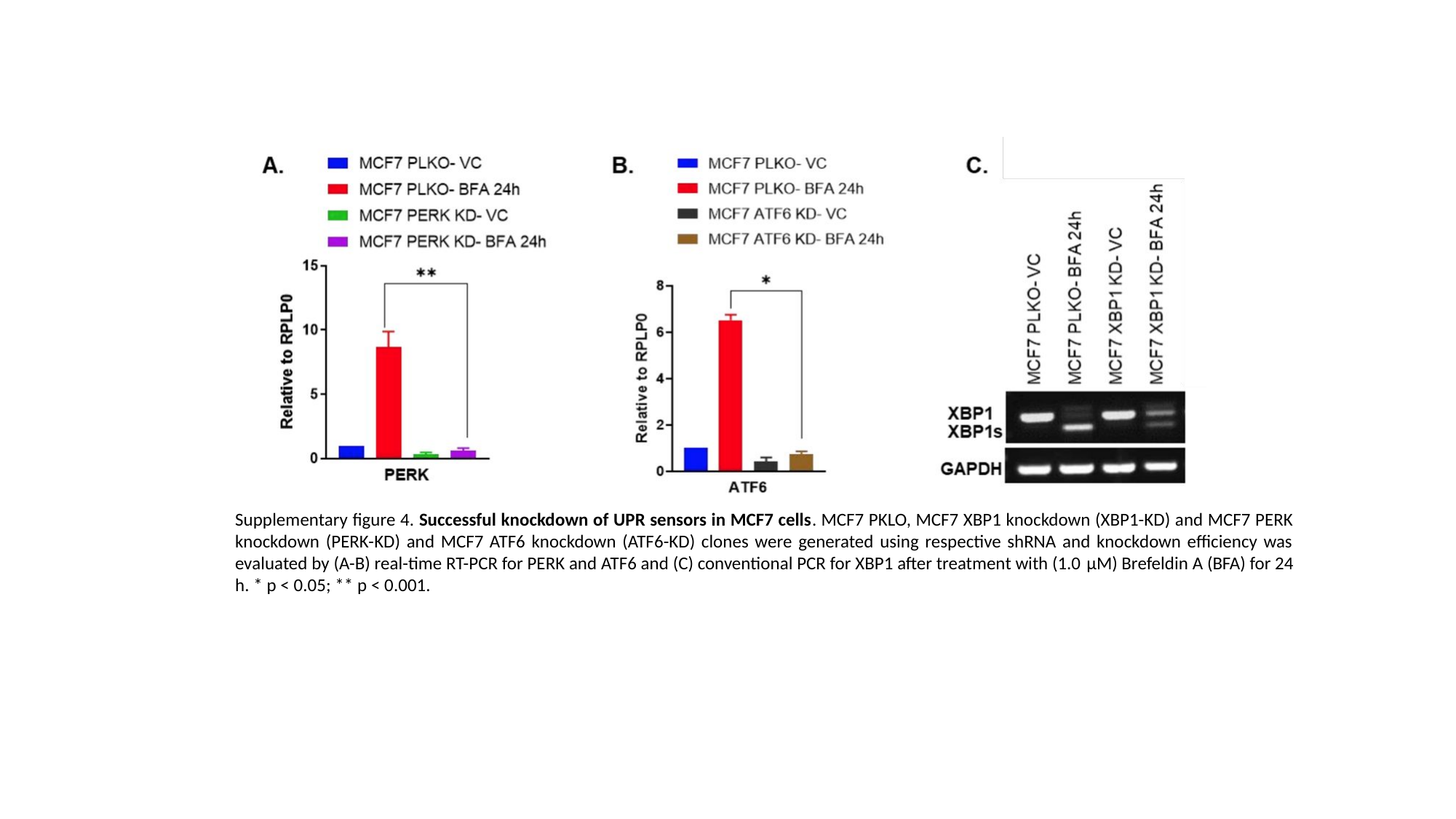

Supplementary figure 4. Successful knockdown of UPR sensors in MCF7 cells. MCF7 PKLO, MCF7 XBP1 knockdown (XBP1-KD) and MCF7 PERK knockdown (PERK-KD) and MCF7 ATF6 knockdown (ATF6-KD) clones were generated using respective shRNA and knockdown efficiency was evaluated by (A-B) real-time RT-PCR for PERK and ATF6 and (C) conventional PCR for XBP1 after treatment with (1.0 μM) Brefeldin A (BFA) for 24 h. * p < 0.05; ** p < 0.001.

## Slide 5
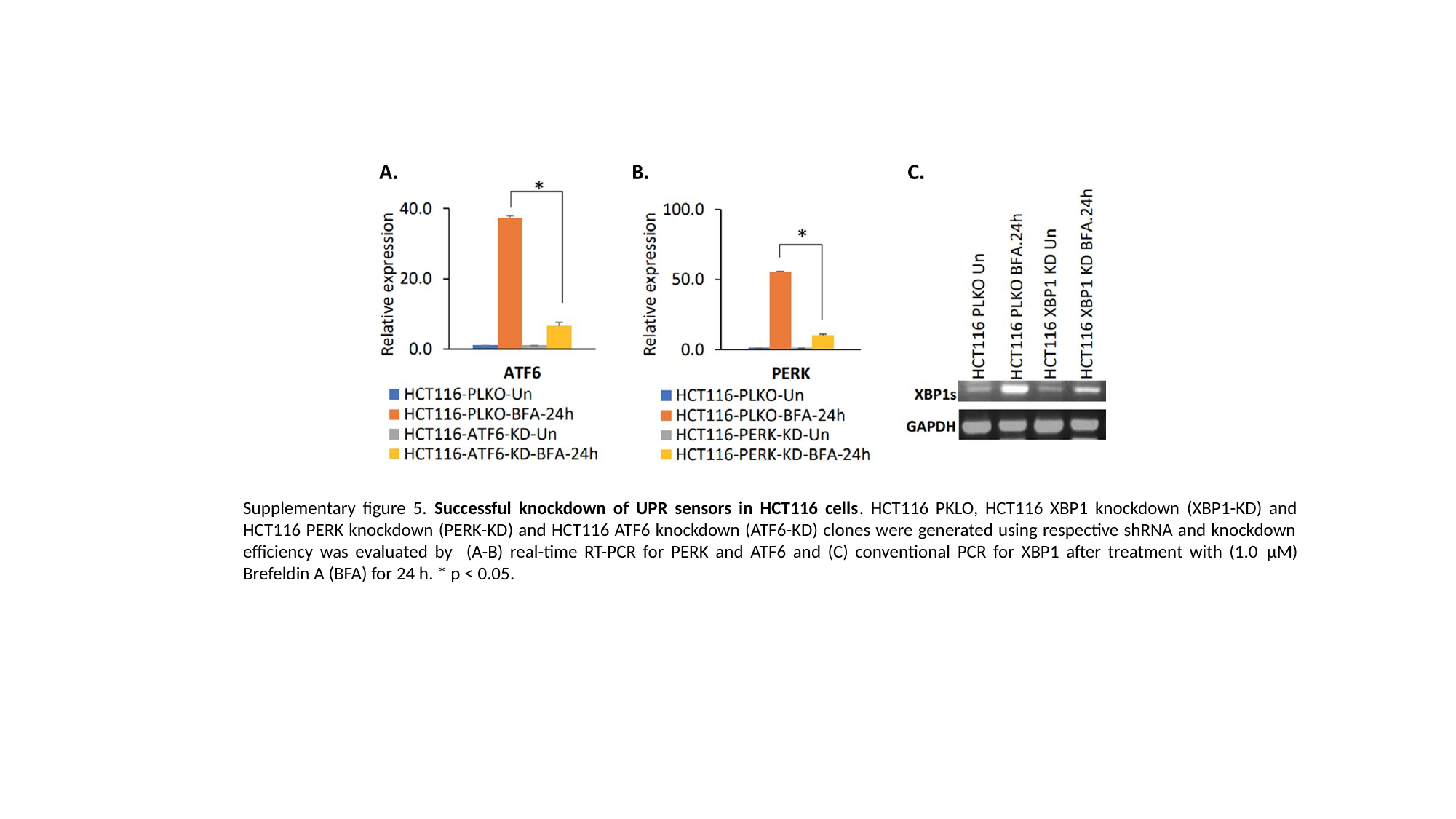

Supplementary figure 5. Successful knockdown of UPR sensors in HCT116 cells. HCT116 PKLO, HCT116 XBP1 knockdown (XBP1-KD) and HCT116 PERK knockdown (PERK-KD) and HCT116 ATF6 knockdown (ATF6-KD) clones were generated using respective shRNA and knockdown efficiency was evaluated by (A-B) real-time RT-PCR for PERK and ATF6 and (C) conventional PCR for XBP1 after treatment with (1.0 μM) Brefeldin A (BFA) for 24 h. * p < 0.05.

## Slide 6
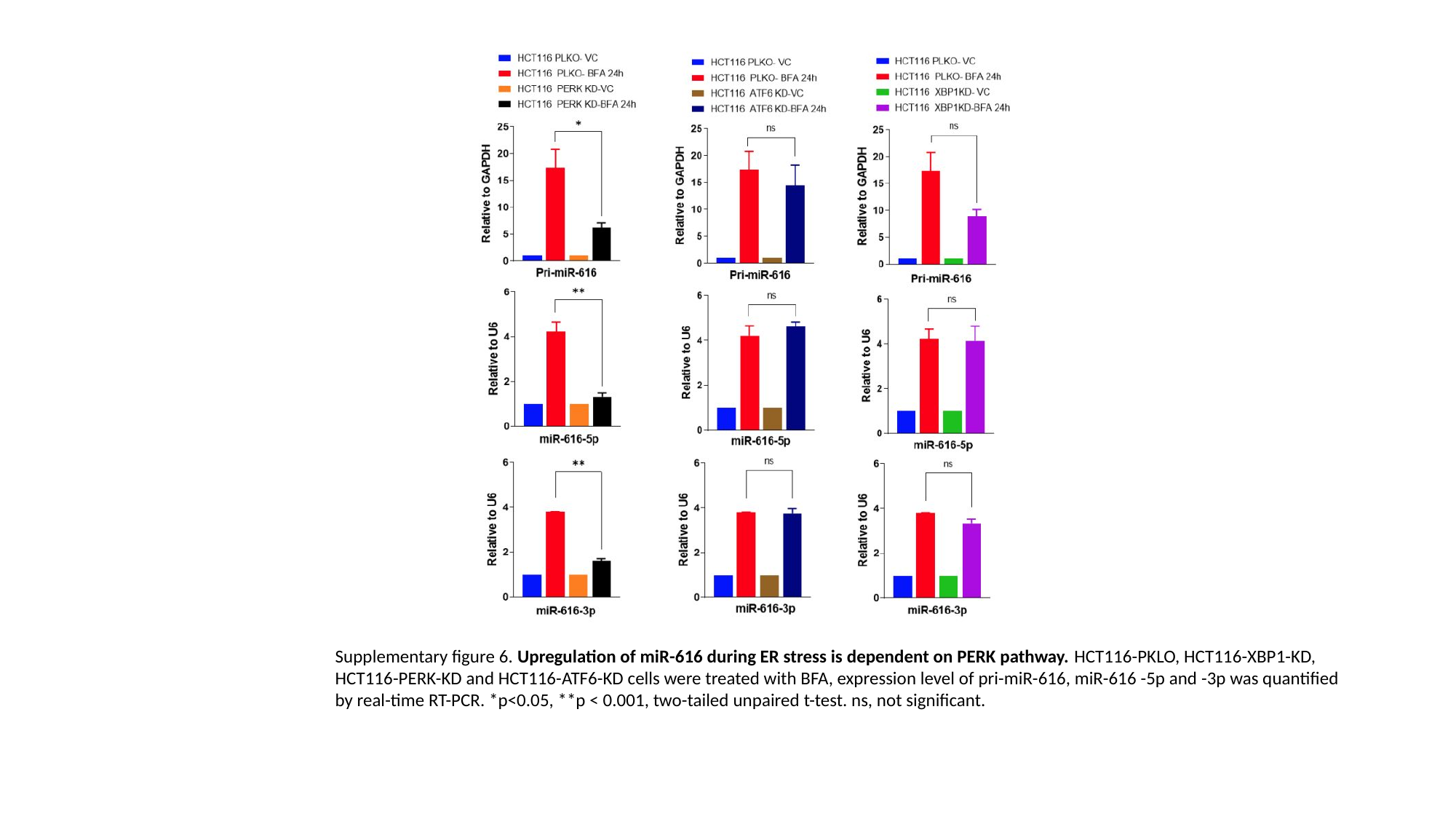

Supplementary figure 6. Upregulation of miR-616 during ER stress is dependent on PERK pathway. HCT116-PKLO, HCT116-XBP1-KD, HCT116-PERK-KD and HCT116-ATF6-KD cells were treated with BFA, expression level of pri-miR-616, miR-616 -5p and -3p was quantified by real-time RT-PCR. *p<0.05, **p < 0.001, two-tailed unpaired t-test. ns, not significant.

## Slide 7
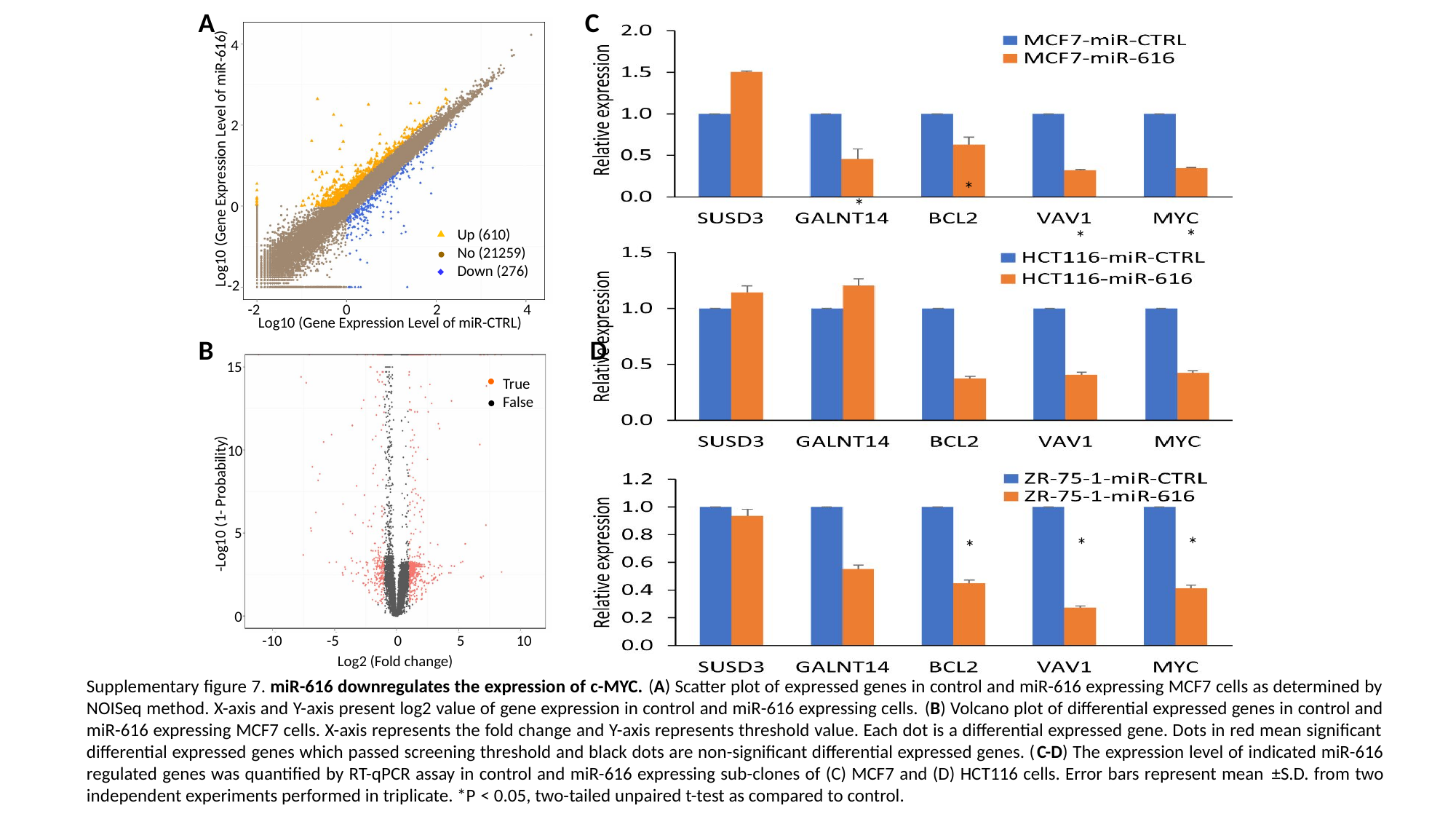

A C
B D
4
2
0
-2
Log10 (Gene Expression Level of miR-616)
Up (610)
No (21259)
Down (276)
-2 0 2 4
Log10 (Gene Expression Level of miR-CTRL)
15
10
5
0
-Log10 (1- Probability)
True
False
 -10 -5 0 5 10
Log2 (Fold change)
 *
 *
 *
 *
 *
 *
 *
Supplementary figure 7. miR-616 downregulates the expression of c-MYC. (A) Scatter plot of expressed genes in control and miR-616 expressing MCF7 cells as determined by NOISeq method. X-axis and Y-axis present log2 value of gene expression in control and miR-616 expressing cells. (B) Volcano plot of differential expressed genes in control and miR-616 expressing MCF7 cells. X-axis represents the fold change and Y-axis represents threshold value. Each dot is a differential expressed gene. Dots in red mean significant differential expressed genes which passed screening threshold and black dots are non-significant differential expressed genes. (C-D) The expression level of indicated miR-616 regulated genes was quantified by RT-qPCR assay in control and miR-616 expressing sub-clones of (C) MCF7 and (D) HCT116 cells. Error bars represent mean ±S.D. from two independent experiments performed in triplicate. *P < 0.05, two-tailed unpaired t-test as compared to control.

## Slide 8
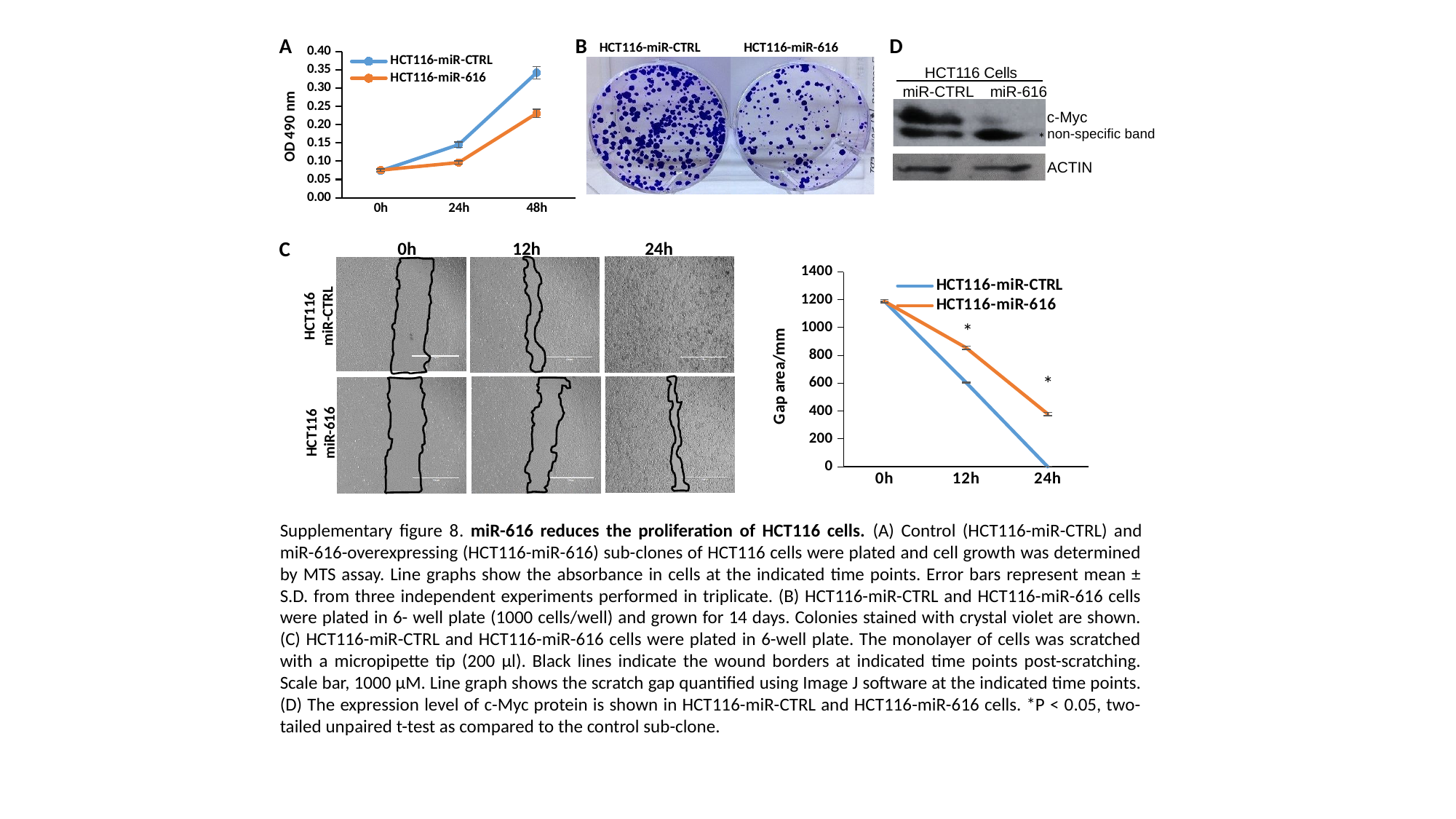

A B D
C
 HCT116-miR-CTRL HCT116-miR-616
### Chart
| Category | HCT116-miR-CTRL | HCT116-miR-616 |
|---|---|---|
| 0h | 0.0736888888888889 | 0.07555555555556 |
| 24h | 0.14533333333333334 | 0.09633333333333 |
| 48h | 0.3424666666666667 | 0.231 |HCT116 Cells
miR-CTRL miR-616
c-Myc
ACTIN
*
 0h 12h 24h
HCT116
miR-CTRL
HCT116
miR-616
### Chart
| Category | HCT116-miR-CTRL | HCT116-miR-616 |
|---|---|---|
| 0h | 1189.0 | 1189.0 |
| 12h | 605.0 | 855.0 |
| 24h | 0.0 | 379.0 |*
*
non-specific band
Supplementary figure 8. miR-616 reduces the proliferation of HCT116 cells. (A) Control (HCT116-miR-CTRL) and miR-616-overexpressing (HCT116-miR-616) sub-clones of HCT116 cells were plated and cell growth was determined by MTS assay. Line graphs show the absorbance in cells at the indicated time points. Error bars represent mean ± S.D. from three independent experiments performed in triplicate. (B) HCT116-miR-CTRL and HCT116-miR-616 cells were plated in 6- well plate (1000 cells/well) and grown for 14 days. Colonies stained with crystal violet are shown. (C) HCT116-miR-CTRL and HCT116-miR-616 cells were plated in 6-well plate. The monolayer of cells was scratched with a micropipette tip (200 µl). Black lines indicate the wound borders at indicated time points post-scratching. Scale bar, 1000 µM. Line graph shows the scratch gap quantified using Image J software at the indicated time points. (D) The expression level of c-Myc protein is shown in HCT116-miR-CTRL and HCT116-miR-616 cells. *P < 0.05, two-tailed unpaired t-test as compared to the control sub-clone.

## Slide 9
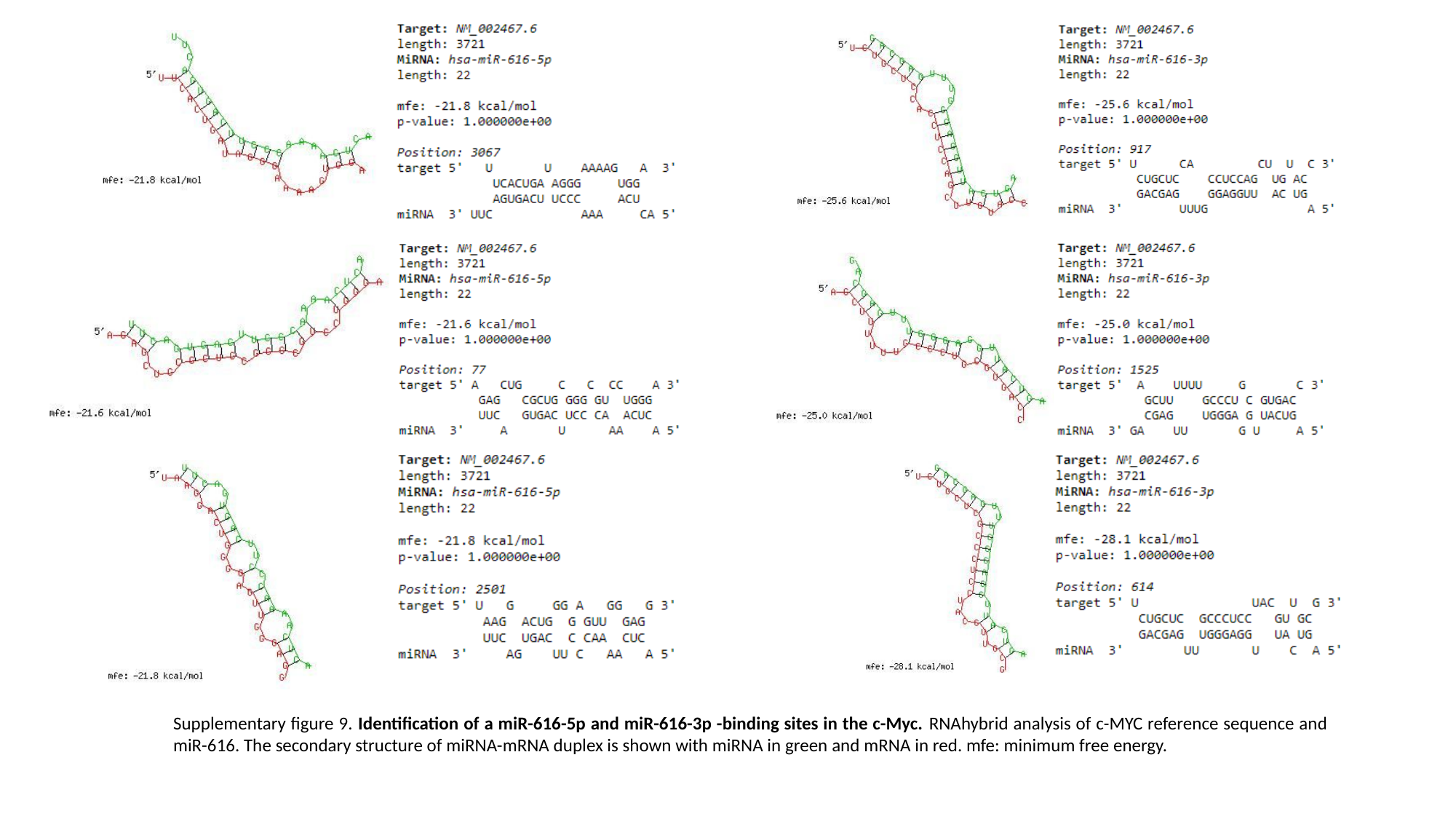

Supplementary figure 9. Identification of a miR-616-5p and miR-616-3p -binding sites in the c-Myc. RNAhybrid analysis of c-MYC reference sequence and miR-616. The secondary structure of miRNA-mRNA duplex is shown with miRNA in green and mRNA in red. mfe: minimum free energy.

## Slide 10
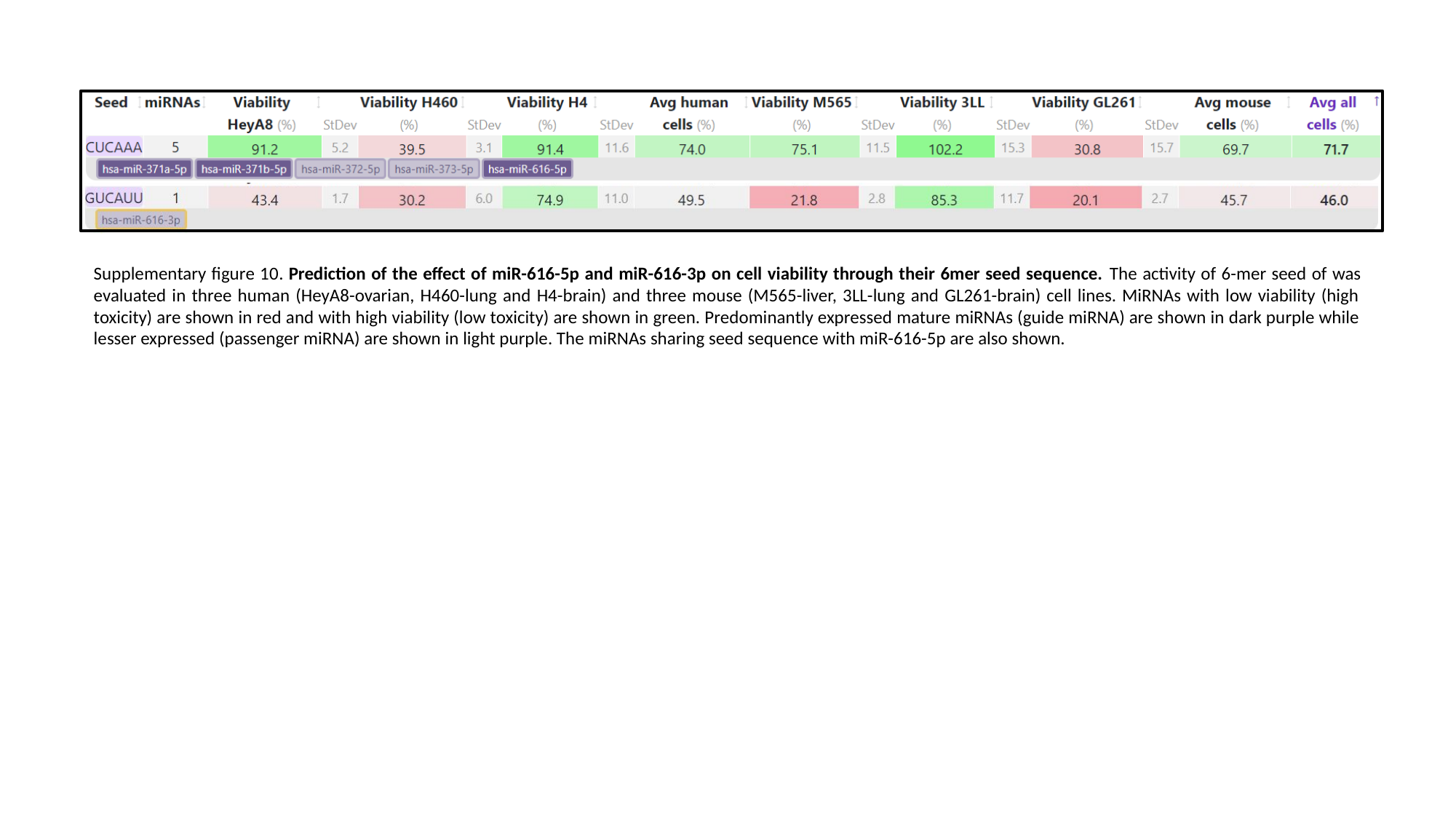

Supplementary figure 10. Prediction of the effect of miR-616-5p and miR-616-3p on cell viability through their 6mer seed sequence. The activity of 6-mer seed of was evaluated in three human (HeyA8-ovarian, H460-lung and H4-brain) and three mouse (M565-liver, 3LL-lung and GL261-brain) cell lines. MiRNAs with low viability (high toxicity) are shown in red and with high viability (low toxicity) are shown in green. Predominantly expressed mature miRNAs (guide miRNA) are shown in dark purple while lesser expressed (passenger miRNA) are shown in light purple. The miRNAs sharing seed sequence with miR-616-5p are also shown.
