# Supplementary material for: UPR-Induced miR-616 Inhibits Human Breast Cancer Cell Growth and Migration by Targeting c-MYC
Source: Int J Mol Sci. 2023 Aug 22;24(17):13034. doi: 10.3390/ijms241713034 (PMC10487498; doi:10.3390/ijms241713034)
Supplement: Supplementary file 1 [file ijms-24-13034-s001.zip › Supplementary methods miR-616 IJMS.pdf]

UPR-induced miR-616 inhibits human breast cancer cell growth and migration by targeting c-MYC

Supplementary Methods

MTS cell proliferation assay - The control and miR-17-92 expressing cells (2×10^3 cells/well) were plated in 96-well plates. After 24 h of culture, for cytotoxicity assay, cells were treated with (1, 10, 50 and 100 μM) Fulvestrant, (1, 10, 100 and 250 μg/ml) 5-F-Uracil, (250, 500, 1000 and 2000 ng/ml) Doxorubicin, (10, 100, 1000 and 2000 nM) Docetaxel and (1, 5, 10 and 50 nM) Bortezomib for 48 h. The 0.02 ml of the MTS solution from CellTiter 96® AQ$_{ueous}$ One Solution Cell Proliferation Assay kit (Promega, Cat # G3582) and PMS (Sigma-Aldrich, Cat # 78830-1G) solution was directly added into each well and incubated at 37°C for 4 h. To prepare MTS + PMS solution, 100 μl of PMS (0.9 mg/ml) solution was added to 1 ml of MTS solution. The absorbance of each well was measured at 490 nm with a 96-well plate reader (Synergy ^{TM} HT, BIO-TEK, Vermont, USA).

High-throughput mRNA sequencing and analysis- RNA-seq was used to determine the mRNA expression profiles in control and miR-17-92 expressing MCF7 cells (four replicates) by BGI Tech solutions, Hong Kong. We obtained an average of 43,802,853 raw sequencing reads and 43,749,166 clean reads (after filtering low quality reads) for each sample. Clean reads were then mapped to reference using HISAT and Bowtie2. The average mapping ratio with reference gene was 82.81% and the average genome mapping ratio was 95.95%. Fragments per kilobase of transcript per million mapped reads (FPKM) method was used to calculate expression level, using the following formula: $FPKM = 10^6C / (NL/10^3)$

For the expression of a given gene A, C is number of fragments that are aligned to gene A, N is the total number of fragments that are aligned to all genes, and L is the number of bases present in the gene A. We used NOISeq method to screen for differentially expressed genes between control and miR-17-92 expressing groups. NOISeq uses sample's gene expression in each group to calculate log2 fold change (M) and absolute different value (D) of control and miR-17-92 expression group to build noise distribution model. For any given gene A, NOISeq computes its average expression "Control_avg" in control group and average expression

"Treat_avg" in treatment group. Next the fold change and absolute different value was analysed using the following formula:

Fold change for gene A, MA=log2 [(Treat_avg)/(Control_avg)]
Absolute different value for gene A, DA=|Control_avg-Treat_avg|

If MA and DA diverge from noise distribution model markedly, gene A will be defined as a differentially expressed gene (DEG). There is a probability value to assess how MA and DA both diverge from noise distribution model. Finally, DEG were identified according to the following default criteria: Fold change ≥2 and diverge probability ≥0.8.

Supplementary Table S1. List of differentially expressed genes in MCF7 miR-616 cells as compared to MCF7 miR-CTL cells.

Supplementary Table S2. List of primers and probes used for PCR.
