# Supplementary material for: UPR-Induced miR-616 Inhibits Human Breast Cancer Cell Growth and Migration by Targeting c-MYC
Source: Int J Mol Sci. 2023 Aug 22;24(17):13034. doi: 10.3390/ijms241713034 (PMC10487498; doi:10.3390/ijms241713034)
Supplement: Supplementary file 1 [file ijms-24-13034-s001.zip › Supplementary Table S2 mir616 IJMS.pdf]

Supplementary Table S2. List of primers and probes used for PCR.

TaqMan qPCR assays (IDT)

Primer	Direction	5'to3' primer sequence
SUSD3	Probe	5'/56-FAM/ACC ACA GCT /ZEN/TCA CC CAG ACC AT/3IABkFQ/3'
	Primer 1	5'-CCT CAA GCA CTT CAA CAA ACC-3'
	Primer 2	5'-GAT CCC AGG GTC CTT GTC-3'
GALNT14	Probe	5'/56-FAM/CCC CAG TG /ZEN/TTC TTG TCC TTT GCA /3IABkFQ/3'
	Primer 1	5'-GAG CTG TGC CTG TCA GTC-3'
	Primer 2	5'-ATG CTA TGT GCT CGA TGT GG-3'
BCL2	Probe	5'/56-FAM/AAC AAA TGC /ZEN/ATA AGG CAA CGA C/3IABkFQ/3'
	Primer 1	5'GCT ATA ACT GGA GAG TGC TGA AG3'
	Primer 2	5'AGT CTA CTT CCT CTG TGA TGT TG3'
VAV1	Probe	5'/56-FAM/CCA TAG ATC /ZEN/TCC CCT CGC CAC CA/3IABkFQ/3'
	Primer 1	5'GTG ACA TCA TCA AGA TCC TTA ACA AG3'
	Primer 2	5'CAG AAT AAT CTT CCT CCA CGT AGT3'
MYC	Probe	5'/56-FAM/TGG GCG GTG /ZEN/TCT CCT CAT GG/3IABkFQ/3'
	Primer 1	5'TCC TCG GAT TCT CTG CTC TC3'
	Primer 2	5'TCT TCC TCA TCT TCT TGT TCC TC3'
GAPDH	Probe	5'-/56FAM/CAG CAA GAG/ZEN/CAC AAG AGA GAG AGA/3IABkFQ/-3'
	Primer 1	5'-AGG GTG GTG GAC CTC AT-3'
	Primer 2	5'-TGA GTG TGG CAG GGA CT-3'
RPLP0	Probe	5'-/56-FAM/CCA GGA CTC/ZEN/GTT TGT ACC CGT TGA T/3IABkFQ/-3'
	Primer 1	5'-GGT GTA ATC CGT CTC CAC AG-3'
	Primer 2	5'-GAA ACT CTG CAT TCT CGC TTC-3'
PERK	Probe	5'-/56-FAM/TTT CTT GAT/ZEN/GGA GAG AGC GGG CTG G/3IABkFQ/-3'
	Primer 1	5'-AGG ACT TGC CTA TGG AAA CC-3'
	Primer 2	5'-CTG TTG CTC CAA GGT GTG AT-3'
ATF6	Probe	5'-/56FAM/AGG ACT G/ZEN/AGG ATA GCA AGA TGA GAA/3IABkFQ/-3'
	Primer 1	5'-AAC TTT AGT CGG CCT GAT GG-3'
	Primer 2	5'-GAA TCA AAG CAA AAG CCT GTC C-3'

Primers used for conventional PCR

Primer Name	Sequence of Primer	Product Size
GAPDH	Forward: 5'-ACC ACA GTC CAT GCC ATC - 3'<br>Reverse: 5'-TCC ACC ACC CTG TTC CTG- 3'	452 bp
XBP1s	Forward: 5'-GGG AAT GAA GTG AGG CCA G- 3<br>Reverse: 5'-TGA AGA GTC AAT ACC GCC A- 3'	XBP1u: 137 bp<br>XBP1s: 111 bp

miRNA Assay Name	Mature miRNA sequence	Assay ID	MirBase ID and accession no.
U6 SnRNA	GTGCTCGCTTCGGCAGCACATATACTAAAATTGGAACGATACAGAGAAGATTAGCATGGCCCCTGCGCAAGGATGACACGCAAATTCGTGAAGCGTTCCATATTTT	001973	
hsa-miR-616-5p	ACUCAAAACCCUUCAGUGACUU	001589	hsa-miR-616-5p MIMAT0003284
hsa-miR-616-3p	AGUCAUUGGAGGGUUUGAGCAG	002414	hsa-miR-616-3p MIMAT0004805
Primary hsa-miR-616	UUAGGUAAUUCCUCCACUCAAAACCCUUCAGUGACUUCCAUGACAUGAAAUAGGAAGUCAUUGGAGGGUUUGAGCAGAGGAAUGACCUGUUUUAAAA	Hs03304633_pri	MI0003629

TaqMan qPCR assays for microRNAs (Applied Biosystem)
